# Honey: A Potential Therapeutic Agent for Managing Diabetic Wounds

**DOI:** 10.1155/2014/169130

**Published:** 2014-10-15

**Authors:** Fahmida Alam, Md. Asiful Islam, Siew Hua Gan, Md. Ibrahim Khalil

**Affiliations:** ^1^Human Genome Centre, School of Medical Sciences, Universiti Sains Malaysia, 16150 Kubang Kerian, Kelantan, Malaysia; ^2^Department of Biochemistry and Molecular Biology, Jahangirnagar University, Savar, Dhaka-1342, Bangladesh

## Abstract

Diabetic wounds are unlike typical wounds in that they are slower to heal, making treatment with conventional topical medications an uphill process. Among several different alternative therapies, honey is an effective choice because it provides comparatively rapid wound healing. Although honey has been used as an alternative medicine for wound healing since ancient times, the application of honey to diabetic wounds has only recently been revived. Because honey has some unique natural features as a wound healer, it works even more effectively on diabetic wounds than on normal wounds. In addition, honey is known as an “all in one” remedy for diabetic wound healing because it can combat many microorganisms that are involved in the wound process and because it possesses antioxidant activity and controls inflammation. In this review, the potential role of honey's antibacterial activity on diabetic wound-related microorganisms and honey's clinical effectiveness in treating diabetic wounds based on the most recent studies is described. Additionally, ways in which honey can be used as a safer, faster, and effective healing agent for diabetic wounds in comparison with other synthetic medications in terms of microbial resistance and treatment costs are also described to support its traditional claims.

## 1. Introduction

Diabetes mellitus (DM) is a progressive and chronic endocrine disorder that primarily results in hyperglycemia (excess glucose in the blood). Globally, diabetes is considered to be one of the major health problems with increasing prevalence. The prevalence of diabetes among all age groups worldwide was 2.8% in 2000 (171 million) and is estimated to increase (4.4%) by 2030 (366 million). At present, 200 million people worldwide are suffering from diabetes and this figure is predicted to increase up to 333 million by the end of 2025. The highest (relative and absolute) increment will occur in developing countries, where the prevalence will rise from 4.2% to 5.6% [1, 2].

Data from large epidemiological studies have indicated that the worldwide incidence of type I DM has been increasing by 2–5% with an approximate prevalence of one in 300 by 18 years of age in the United States [3]. The global prevalence of type 2 DM (T2DM) has shown rapid growth over the past few decades. According to the U.S. National Health and Nutrition Examination Survey, more than 40% of U.S. adults have diabetes or are prediabetic, which has doubled (from 4% to 8%) in the past 40 years [4, 5]. By the year 2025, it is estimated that there will be 40 million diabetic patients in China and India alone [6]. According to the statistics of the International Diabetes Federation (IDF), two individuals will develop diabetes and another two will die of diabetes-related conditions every 10 sec around the world [7]. Therefore, diabetes has become a very serious public health problem that causes a socioeconomic burden in many countries.

Diabetic patients tend to suffer from lower extremity complications including peripheral neuropathy, arterial disease, vascular problems, and ulcerations that contribute to the occurrence of diabetic foot infections. Approximately 25% of diabetic patients have a higher reported lifetime risk of developing foot complications [8], and foot ulceration is the most common with an estimated annual incidence from 25 to 80% [9]. Most foot ulceration ultimately turns into diabetic gangrene especially if left untreated, contributing to approximately 80% of lower limb amputations [10–16]. More than 50% of diabetic wounds can exponentially increase the risk of below-knee amputation [17–19], which significantly enhances mortality in addition to contributing to a poor quality of life with enormous social, psychological, and economic consequences [13, 20–23]. The majority of diabetic foot ulcerations involve the toes [24]. If proper treatment is not provided in due time, the amputation of the affected bone becomes unavoidable [25].

Wound healing is an intricate process involving skin (or another organ tissue) repair following injury [26]. In addition to being complex, it is also a dynamic process in which devitalized and missing cellular structures and tissue layers must be replaced. Despite recent advances in antimicrobial therapy, diabetic foot wounds remain a serious problem. Treating foot ulcers is protracted, intensive, and associated with high costs. For these reasons, various treatment approaches have been adopted including the use of topical wound-care therapies [27].

Although numerous topical and systemic agents have been used either solely or in combination to eradicate infections, many have been eliminated because of resistance. These agents have led to the emergence and subsequent rapid overgrowth of resistant bacterial strains, drug side effects, and organ-specific toxicity [28–30]. Diabetic wound infections caused by drug-resistant organisms are now becoming more common and have increasing resistance to commonly used antibiotics, ultimately leading to increased costs, morbidity, and mortality [31, 32]. With an increasing frequency of antibiotic-resistant pathogens, modern medicine directs attention to natural products with antimicrobial activity for clinical practice [27].

Honey is a collection of nectar from many plants, and this nectar is processed by honey bees. This natural product is well known for its high nutritional and prophylactic medicinal value [33]. Honey has potent antibacterial activity and is effective in preventing and clearing wound infections [23, 34]. It has been used as a wound care product, and its use as a wound healing agent was reported for treating venous leg ulcers [35, 36], burns [37, 38], chronic leg ulcers [39], pressure ulcers [40, 41], and exit sites for central venous catheters [42].

Honey has several natural substances that contribute to its antimicrobial activity including an osmotic effect, a naturally low pH, and the production of hydrogen peroxide [43–45]. Recent investigations have revealed that honey combats antibiotic-resistant strains of bacteria and prevents bacterial growth even when wounds are heavily infected [46, 47]. Furthermore, because honey is a natural product, it does not induce microbial resistance, even if the honey is unsuccessful in killing the microbes [48].

The objective of this review is to illustrate how and why honey should be considered as one of the best complementary and alternative medicines in diabetic wound management. It also provides scientific evidence to support the traditional use of honey in treating diabetic wounds.

## 2. Honey in Association with Diabetic Wounds

Although DM is almost harmless if controlled, the state of abnormally high blood glucose levels associated with the disease can lead to some serious complications. Although diabetic wounds are similar to wounds in normal patients, the healing process is different from that of other wounds. The most crucial part of diabetic wounds is that the healing process is notoriously slow. Hypoxia occurs in diabetic wounds, and it is caused by early inflammatory responses and a high load of reactive oxygen species (ROS) [49] induced by hyperglycemia in diabetic patients [50]. The formation of advanced glycation end-products (AGEs) under hyperglycemic conditions and interactions with their receptors (RAGE) are also associated with impaired wound healing among diabetic patients [51]. Several dysregulated cellular functions involved in diabetic wounds (defective T-cell immunity, defects in leukocyte chemotaxis, phagocytosis, bactericidal capacity, dysfunctions in fibroblasts and epidermal cells) were attributed to inadequate bacterial clearance and delayed or impaired repair in individuals with diabetes [52].

In addition, slow wound recovery generally increases treatment costs. It has been reported that the total direct costs for healing infected diabetic foot ulcers that do not require amputation are approximately $17,500, whereas the costs for lower-extremity amputations are between $30,000 and $33,500 depending on the level of amputation [53]. Because of these high costs, scientists have been searching for a cheaper, naturally sourced remedy for diabetic wounds that is efficacious. Honey is a potential candidate because it is easily available and natural.

## 3. The Effects of Honey Antioxidants on Diabetic Wounds

Hydroxyl radicals and hypochlorite anions are formed from superoxide anions produced by activated polymorph nuclear neutrophils (PMNs) at the wound site, and they are considered to be important factors in impaired wound healing. The superoxide anion may also react with the nitric oxide that is produced by macrophages to form peroxynitrite, a third strong oxidant that damages the surrounding tissues [54, 55]. Over many years, honeys from different parts of the world have been shown to be one of the highest potential natural products in which phenolics, flavonoids, ascorbic acids, and some enzymes (glucose oxidase and catalase) serve as potent antioxidants [56–59]. The antioxidants found in honey work on wounds through two means. First, the antioxidants fight against microorganisms and decrease infections at the site of the wound [60–62]. Second, the antioxidants reduce reactive oxygen species (ROS) and inflammations caused by the wound and aid in the healing process [62–65]. The combined antioxidant effects may have contributed to some successful clinical evidence from diabetic wounds showing more effective wound recoveries upon topical honey applications (Table 1).

## 4. Antimicrobial Activity

The broad spectrum antimicrobial activity of honey has been demonstrated in various studies. Honey reportedly exerts both bacteriostatic and bactericidal activities [66, 67]. Because of the emergence of antibiotic-resistant microorganisms in diabetic wound treatment, the use of honey as an effective wound treatment is increasing because it can markedly inhibit the activities of wound-isolated microorganisms (Table 2). Some of the properties of honey (acidity, osmosis, hydrogen peroxide, and nitric oxide) that contribute to its antimicrobial activities against diabetic wounds are discussed below.

### 4.1. Honey Acidity

Honey is characteristically found at an acidic pH ranging between 3.2 and 4.5 [68]. The acidity of honey is primarily caused by the presence of gluconolactone or gluconic acid, and approximately 0.23–0.98% (1.8–7.5 mmol/kg) [69] is formed through the action of a glucose oxidase enzyme produced by the bees. According to Al-Waili and Saloom [70], honey acidity is considered to be one of the factors that contributes to its antimicrobial activity. The glucose content of honey and its acidic pH may aid in bacterial killing by macrophages [71]. In addition, because of its acidic nature, honey can prevent microbial biofilm formation and cross contamination [72]. The acidity of honey creates an environment that facilitates the release of oxygen from the hemoglobin that is required for newly growing cells and the stimulation of white blood cells [73]. It is possible to increase the oxygen release rate from hemoglobin by lowering the wound pH via honey application, thus increasing tissue granulation [74] and improving the wound healing rate in diabetic patients [75]. Moreover, acidifying a wound through honey application can potentially reduce the protease activity [75] and provide a suitable environment for increasing fibroblast activity [67, 75], consequently promoting wound healing.

A previous study investigated the effects of Manuka honey dressing following two weeks of application on a nonhealing ulcer by collecting measurements of the change in wound surface pH and the ulcer size. A statistically significant reduction in the wound pH and size was observed. When the wound was in an environment with a pH ≥ 8.0, the size did not decrease. However, the pH was remarkably reduced to ≤7.6 with a 30% decrease in the wound size upon honey application [76], indicating that the acidic condition created by honey favors wound healing.

### 4.2. Honey's Osmotic Effects

Honey that contains <20% water is hyperosmolar [77]. By being hyperosmolar, honey creates an unfavorable environment for the growth and survival of microorganisms [78]. High osmolarity substrates such as honey, glucose, and sugar pastes can inhibit microbial growth because water molecules are chemically tied to the sugar molecules, thus creating a nonconducive environment for organism survival, leading to death [46]. Therefore, the hyperosmolar condition created by honey is also important for treating infections because it prevents the growth of bacteria and encourages rapid wound healing [79]. Sugar has been shown to accelerate wound healing in many patients with wounds, burns, and ulcers [80].

The sugar content of honey is purportedly responsible for its antibacterial activity, which is contributed entirely by the osmotic effect [81–84]. Hyperosmolar substances tend to draw fluid into the wound area to make a viscous solution, thus providing a protective layer against cross contamination [67]. Therefore, a highly osmolar solution, that is, honey, can be safely employed for diabetic wound treatments. However, only undiluted honey is sufficient for preventing microbial growth because its osmotic inhibition is lost when honey becomes diluted by wound exudates [66]. In addition, the osmotic action on bacteria is limited to the wound surface only, whereas other antibacterial factors can diffuse into wound tissues. However, the potency of the additional antibacterial factors varies as much as one hundredfold from honey to honey [85].

### 4.3. Hydrogen Peroxide

The hydrogen peroxide (H_2_O_2_) that is found in honey is steadily produced by oxidation from the glucose oxidase enzyme (which is secreted into nectar from the hypopharyngeal gland of bees), and it is also a potent antibacterial agent [43]. Glucose oxidase is inactive in concentrated honey solutions (because of the low pH), but upon honey dilution, it is activated and produces H_2_O_2_ [86, 87]. The production rate of H_2_O_2_ by glucose oxidase varies notably in honey and increases disproportionally depending on the degree of honey dilution [88]. Even when honey is applied to the wound area, the rate of H_2_O_2_ production, destruction, and dilution by exudates in a wound varies over time [89]. The H_2_O_2_ produced by honey is not cytotoxic because its H_2_O_2_ concentration is approximately 1000 times lower than that of the 3% solution commonly used as an antiseptic [90]. The low concentration of hydrogen peroxide may act as a “messenger” in healing promotion and may stimulate both fibroblasts and epithelial cells [67]. H_2_O_2_ reportedly stimulates fibroblast proliferation* in vitro* and angiogenesis* in vivo* [91]. Interestingly, the presence of high antioxidant levels in honey could reportedly confer protection to wound tissues from oxygen radicals that may be produced by H_2_O_2_ [92].

An experimental study using zebra fish has revealed a novel mechanism of early leukocyte migration to wounds as a result of the concentration gradient created by H_2_O_2_ [93]. Neutrophils release bactericidal reactive oxygen species and H_2_O_2_ kills bacteria and prevents infection. Macrophages arrive at the wound in response to environmental stimuli and release vascular endothelial growth factor (VEGF), an angiogenic factor that is crucial in the wound healing process. The released H_2_O_2_ increases macrophage VEGF through the oxidant induction of the VEGF promoter. This oxidant stimulation can be mediated by activated neutrophils [94]. Although only low levels of H_2_O_2_ accumulate in diluted honey, this is still an effective antimicrobial system because of its continuous production.

H_2_O_2_ has also been found to be more effective when supplied by continuous generation from glucose oxidase as opposed to when it is added as a bolus [95]. A substantial correlation has been found between the level of endogenous H_2_O_2_ and the extent of bacterial growth inhibition by honey [87]. In Canada, it was even suggested that the antimicrobial activity in some honeys depends on the endogenous H_2_O_2_ content. Based on a broth microdilution assay, the antibacterial activities of 42 Canadian honeys against the two bacterial strains* Escherichia coli* (ATCC 14948) and* Bacillus subtilis* (ATCC 6633) were analyzed. The findings indicated that all honey samples exhibited antibacterial activity with higher selectivity against* E. coli* than* B. subtilis.* Furthermore, antibacterial activity was correlated with H_2_O_2_ production in honey [96]. According to Brudzynski et al. [97], endogenous H_2_O_2_ inhibits* E. coli* in a concentration-dependent manner, although its minimum inhibitory concentration (MIC_90_) value was twofold higher (at 2.5 mM) than those of exogenous H_2_O_2_ (1.25 mM). Therefore, the H_2_O_2_ that was liberated from honey could both control wound infection and improve wound healing.

### 4.4. Nitric Oxide

Nitric oxide (NO) plays an important role in the immunological response, inflammatory response, cell movement, and killing mechanisms of bacteria and viruses and also supports different types of organ-related functions. NO is very active in the proliferative stages during wound healing in patients [98, 99]. Nitric oxide is able to reverse healing impairment in diabetic patients [100]. NO end products are known to be present in honey, and the concentration of these metabolites varies depending on the honey type [101]. This variation most likely contributed to the fact that honey antimicrobial activity also varies depending on the honey type or origin [102]. Moreover, these end products are increased by honey in various biological fluids such as urine, saliva, and blood plasma [103, 104].

The presence of NO metabolites in honey as well as the increased production of NO products by honey in different body fluids improves wound healing and provides the antimicrobial and immunoregulatory actions of NO [70, 101]. Furthermore, increased NO production from honey could explain the various effects of honey on immunity, bacterial infections, and wound healing [101, 103, 104]. Thus, the NO present in honey and NO-derived end products could be other potent ingredients that could help patients recover from diabetic wounds.

## 5. Managing Wound Debridement

Debridement is a very crucial process that facilitates the diabetic wound healing process. During debridement, old dead cells or tissues are removed by mechanical, chemical, surgical, or autolytic means. There are several mechanisms through which honey facilitates the rapid debridement of diabetic wounds and aids in healing. Honey contains protease enzyme that induces wound tissues to start autolytic debridement (self-digestion) [66]. Honey employs its intense osmolytic power to draw out lymph fluid from the wound tissue, thus creating the moist environment necessary for autolytically removing dead, damaged, or infected wound tissues. This mechanism ensures a continuous supply of proteases at the edge of the wound area and the overlying necrotic tissue. With this combined action, honey removes debris and effortlessly removes slough and necrotic tissue without any feeling of pain [66, 105].

The presence of H_2_O_2_ in honey also plays an important but indirect role by activating proteases during debridement. There are two processes through which protease can be activated during wound healing. First, H_2_O_2_ activates the inactive matrix metalloproteases in connective tissue into active protease [106]. Second, it blocks an inhibitor molecule that is present in diabetic wound tissue (which is responsible for inactivating neutrophil serine protease) and makes protease active [66]. Although wound healing impairment is reportedly related to high protease activity, the amount of protease activity in honey is highly regulated [107] because honey's anti-inflammatory properties can prevent excessive protease activity [66]. Because honey is a balanced natural resource for wound healing through proper debridement, it can be said to be a good natural diabetic wound healer.

## 6. Controlling Wound Odor

Honey has the potential ability to minimize offensive-smelling wounds through its strong osmotic action, which draws exudates and lymph fluid from the wound out towards the surface to add the moisture needed for autolytic debridement [89, 108]. For example, a decrease in wound odor has been reported during the treatment of diabetic foot and leg ulcers [67]. Honey can exert its antimicrobial action both* in vivo* and* in vitro* against odor-producing bacteria, thus reducing their presence in wounds and consequently controlling malodor. Based on previous studies, honey can deodorize wound odor through two mechanisms. First, the presence of some anaerobic bacteria such as* Bacteroides* spp.,* Peptostreptococcus* spp., and* Prevotella* spp. is documented to produce malodor. Second, wound odor is produced by the creation of amino acids through the decomposition of serum, tissue proteins, and dead cells by bacteria.

Honey acts by providing an abundance of glucose as a substrate in preference to amino acids for bacterial metabolism [41]. Therefore, glucose is converted to lactic acid by bacteria in the presence of honey instead of the malodor-producing ammonia, amines, and sulfur compounds typically produced by the metabolism of amino acids [41, 66, 67, 105]. Therefore, using honey on diabetic wounds can be very promising instead of using other synthetic products.

## 7. Honey Minimizes Scar Formation

The free radicals formed by excessive or prolonged inflammation can stimulate fibroblasts to produce a hypertrophic scar made of collagen fibers. Hypertrophic scars can be difficult to counteract during wound healing, and they can be alleviated by honey [66]. According to Vijaya and Nishteswar [73], honey stimulates epithelial cell growth at the skin level and produces soft, smooth, and regular scar surfaces in 80% of cases following complete healing. Topham (2002) reported [109] three potential mechanisms behind scarless healing when honey is applied to wounds as follows: (1) the production of hyaluronic acid from glucose suppresses the formation of fiber-forming collagens; (2) attaching sugar to collagen changes its structure and suppresses its activity; and (3) glucose creates an environment in the wound area that directs wound healing proteoglycans to act without producing excessive amounts of collagens.

## 8. The Role of Nutrients in Honey

The primary problem with diabetic wounds is poor or delayed healing. Healing problems are caused by the peripheral arterial diseases and peripheral neuropathy that can occur with diabetes, in which small blood vessels in different parts of the body, especially in the extremities (hands and feet), tend to be narrower, thus reducing the blood circulation to those areas. A lack of circulation in the extremities can result in a reduced supply of oxygen and nutrients to body tissue and nerves, and a normal supply is necessary for healing. Over time, the nerves in these areas may become damaged, decreasing the sensation of pain, temperature, and touch, making patients more vulnerable to injury.

Honey contains defined substances such as glucose, fructose, sucrose, minerals, vitamins, antioxidants, amino acids, and other products. The natural composition and actual proportion of each substance in honey may play a significant role in its mechanism of action and potency [75]. According to Molan [78], the presence of large quantities of assimilable sugars, vitamins, amino acids, and trace elements in honey contributes to its potential in stimulating tissue growth. A study by Viljanto and Raekallio [110] showed that there was an association between topical applications of nutrition to wounds and increased growth of granulation tissue. Molan [66] further noted that honey helps to stimulate angiogenesis and thereby increases oxygen and nutrients to the wound area for better wound healing. Nevertheless, more investigations are needed to identify the presence of other natural nutritional components in honey that may contribute to its wide biological and therapeutic effects on diabetic wounds.

## 9. Inflammation Control

Although inflammation is a vital part of normal responses to infection or injury from wounding, excessive or prolonged inflammation can obstruct diabetic wound healing or even cause further damage to the wound tissues [66]. Suppressing the inflammation and its associated pain in the wound area with honey reduces vasodilatation. This suppression results in reduced edema and exudates with positive effects on healing. The pressure created inside tissues from edema prevails through the blood flow of oxygen and nutrients through the capillaries [111], which are required for leukocytes to combat infections and fibroblast multiplication for connective tissue synthesis [66, 105]. Thus, the anti-inflammatory effects of honey are crucial in treating diabetic wounds because they reduce edema and its associated pain and improve microcirculation with more oxygen and nutrients, leading to tissue repair [112].

According to Halliwell (1995), another consequence of excessive inflammation is ROS overproduction in tissues from phagocyte activity during the inflammatory process [113]. Being very reactive by nature, oxygen free radicals lead to tissue damage as a result of the breakdown of proteins, nucleic acids, and lipid components of cell membranes, and thus they prevent healing. The anti-inflammatory effects of honey can reduce ROS formation and prevent tissue destruction [114]. Although the mechanism behind honey's anti-inflammatory activity is not well documented, a number of studies have supported its anti-inflammatory effects by showing that honey was able to control both acute and chronic inflammation [90]. Examples of honey's anti-inflammatory effects include (1) decreased amounts of inflammatory cells in histological studies of honey-treated biopsy specimens [66, 105] and (2) the ability of honey also to alter the activity of immunocompetent cells in the wound [67].

Honey also exerts significant actions on both innate and adaptive immune regulation. The stimulation of cytokine production (tumor necrosis factor alpha, interleukin- (IL-) 1*β*, and IL-6) by monocytes [115] and the induction of B and T lymphocyte proliferation [116] are directed by honey. A minimum concentration of honey is responsible for the active proliferation of peripheral blood B and T lymphocytes in addition to the activation of phagocytes in cell culture. The induction of proinflammatory cytokines by honey can also potentially activate immunological response to infections [115]. In addition, honey supplies glucose that is critical for the “respiratory burst” in macrophages that is needed to generate H_2_O_2_ and provides glycolysis substrates for energy production in macrophages [66]. Thus, honey acts as an effective agent to prevent diabetic wound inflammation and microbial infections.

## 10. Benefits and Risks of Using Honey over Other Topical Wound Healing Agents

Honey is not completely free from adverse effects. For example, there has been a report on “peppery” sensation when honey is applied to ulcers in a patient [39]. It is plausible that the low pH and high organic compounds in honey may contribute to the stinging sensation especially in some patients with more sensitized nerve endings. However, patients with neuropathic diabetic foot ulcers may be free from this experience due to lack of sensation. Besides the stinging sensation, honey also poses a small risk of wound infection as it may contain some clostridial spores. However, this risk can be reduced by using gamma-irradiated honey which can kill the spores while maintaining honey's antibacterial activity [78]. Nevertheless, to our knowledge, there has not been a single occurrence of wound infection contributed by clostridial spores with the topical application of honey in approximately 2000 cases reported so far. Although there may be some toxic effects from the ingestion of poorly handled honey [117], there have not been any documented toxic effects associated with the topical application of honey on diabetic wounds in comparison with the risk of using other conventional wound healing therapies (Table 3). Besides these few limitations, many studies reported honey as a nontoxic, nonallergenic, nonirritating healing agent with no cytotoxic effects [108, 118, 119]; it is a safe, cheap, and effective healing agent [120, 121].

## 11. Clinical Evidence

Honey has been used as an orthodox alternative medicine to treat wounds for millennia [122]. Records from ancient Greece, Egypt, the Ayurveda of India, Hippocrates, Aristotle, and the Qu'ran all refer to the healing effects of honey [123], and it has now been rediscovered through rational clinical evidence. Table 1 summarizes the most recent (10 years) evidence of successful honey treatments against diabetic wounds from different parts of the world.

There have been reports of some case studies and clinical and randomized controlled trials which provide considerable evidences indicating the effectiveness of honey in wound healing. A study was conducted to determine whether honey (L-Mesitran) and silver-impregnated dressings are cytotoxic to human skin keratinocytes and dermal fibroblasts* in vitro*. The honey-based product showed excellent cytocompatibility with tissue cell cultures when compared with the silver dressing [119].

Efem [118] conducted a clinical trial using honey dressings to treat patients (*n* = 59) having recalcitrant wounds and ulcers of various etiologies. From this number, even though 47 of the patients had previously been treated with conventional treatments by using commercial wound dressings or antibiotics (both systemic and topical), they showed no signs of healing. Following topical application of honey however, the majority (58 of the 59 patients involved) have shown remarkable improvement indicating the effectiveness of honey application for wound healing.

Dunford and Hanano [124] conducted a prospective, nonrandomized study to compare the effects of honey treatment on 40 patients with venous leg ulcers that failed to heal following 12 weeks of compression therapy. Significant reduction in ulcer pain and size with prompt deodorization were reported after honey treatment, further indicating its effectiveness.

In Greece, Manuka honey-impregnated dressings significantly reduced the healing time and provided rapid disinfection of neuropathic diabetic foot ulcers in type 2 diabetic patients when compared to conventional dressings [125]. In Turkey, a five-week randomized clinical trial was conducted to compare honey dressings (*n* = 15) versus an ethoxy-diaminoacridine plus nitrofurazone dressings (*n* = 11) on pressure ulcer healing. Patients who were treated by honey dressing had significantly better Pressure Ulcer Scale for Healing tool (PUSH tool) scores when compared to subjects treated with the ethoxy-diaminoacridine plus nitrofurazone dressing [41].

In Nigeria, 59 patients with wounds and ulcers, most of which had failed to heal with conventional treatment, were treated with unprocessed honey. The majority showed remarkable improvement following topical application of honey [118].

In Malaysia, the effects of honey dressing were compared with a controlled dressing group (povidone iodine followed by normal saline) for 30 Wagner's grade II diabetic foot ulcers in a prospective study. The authors concluded that honey dressing was a safe, alternative dressing for Wagner's grade II diabetic foot ulcers [126].

Some studies showed evidence that combination of honey with other compounds can be beneficial for wound healing. In Jordan, a study reported that the use of honey/normal saline dressing is more effective in reducing the healing time, cost of hospital stay, rate of amputation and irritation due to dressing material in diabetic foot ulcers as opposed to iodine/hydrogen peroxide dressing [127].

In a case series study, combined, noncontact, low-frequency ultrasound and topical application of medical honey effectively reduced wound dimensions, hastened wound closure, promoted cleansing of the wound bed, and stimulated wound healing in patients with chronic and delayed healing wounds of various etiologies and anatomical locations [128].

In Saudi Arabia, mixtures of Acacia honey,* Commiphora molmol* (Myrrh), and* Nigella sativa* (Black seed) showed effective antibacterial activities on clinical isolates from diabetic foot wounds [129]. However, they recommended that further research is needed to determine the most effective combination of these natural products in a clinical setting. In Saudi Arabia, a randomized controlled study revealed that a combination of conventional treatment for diabetic foot ulcers with Manuka honey-impregnated dressings is superior compared to using conventional treatment alone in controlling wound infection, in promoting complete healing process, and in decreasing the rate of minor amputations [130].

In Egypt, a deep wound with tissue loss in the right foot of a 65-year-old male patient with diabetes mellitus was successfully treated with a paste (800 mg bee propolis, 50 g myrrh, mixed together in honey) which healed following four weeks of usage [131].

## 12. The Acceptance of Honey among Patients with Diabetic Wounds

Honey has attracted attention from patients because of its cost effectiveness and as an alternative wound treatment option that has been applied since ancient times [132]. In rural communities, there was a positive response towards local honey as the treatment of choice among patients [133]. Because honey is sticky, diabetic patients may feel discomfort when applying honey to their foot ulcers. A review of 40 patients treated with antibacterial honey for venous ulcers revealed both positive outcomes and high patient acceptance [124]. Another review of 34 patients using honey for diabetic foot ulcers showed positive outcomes with encouraging patient acceptance [133]. Another study reported “patient comfort” levels as high for 88% with honey wound gel applications and 93% with honey alginate [134]. These studies showed no local or systemic atopic reactions to honey. Therefore, honey can be suggested for use as a safe and satisfying healing agent when applied topically to diabetic wounds.

## 13. Guidelines for Honey Application on Wound

Only good quality honey produced specifically for wound care and accepted by the regulatory authorities should be used. Some examples of medical grade honey with standardized antibacterial activity for use in wound management are* Apiban* (Apimed: Cambridge, New Zealand),* Woundcare* 18+ (Comvita: Te Puke, New Zealand), and* Medihoney* (Capilano: Richlands, Queensland, Australia) [66].

Heating of honey above 37°C should generally be avoided since its enzyme content is easily destroyed by exposure to both heat and light [78]. In addition, honey should be stored in a cool place (approximately 20°C) and the storage containers should be made of either amber glass bottles or sachet of aluminum foils to protect honey from light. It is suggested to avoid storage of honey in plastic containers as plasticizers may leech from plastic and contaminate the honey [135].

The type of honey dressing product should be based on the wound type. The alginate honey dressing is especially attractive for being flexible, simple application, nonadherent to the wound surface and is less painful upon removal [136]. The frequency of dressing change is generally determined by the amount of exudates. However, no evidence is available to suggest the optimum frequency of dressing change needed.

To prevent contamination, the outer dressing must be changed whenever it is moist with exudate. When the amount of drainage decreases, the dressing can be left on for longer periods (4–7 days) which eventually reduces the frequency of dressing changes [136].

It has been reported that honey should be evenly applied on the dressing pad rather than directly onto the wound. Eddy et al. [77] suggested applying honey from 1 to 4 times daily. Nevertheless, the required dosage of honey on the wound depends on the amount of exudates present; the beneficial effects of honey will be reduced if honey is diluted by large amount of exudates. On the other hand, deep wounds require larger amounts of honey to exert antibacterial activity effectively. Therefore, dressings that hold sufficient honey in the wound area are useful to be therapeutically effective.

Honey should be immediately applied on the wound for better outcome as well as to reduce the risk of microbial contamination in honey. In addition, maximum coverage of inflamed wound areas by honey with highest contact is recommended. In case of using nonadherent dressing, it should be porous enough to allow the diffusion of honey components into the wound [137].

To debride hard eschar, dressings soaked in diluted honey (a mixture of 1 volume of honey with 3 volumes of saline) can be applied to allow better diffusion of honey until debridement is achieved [66].

## 14. Future Directions

Honey is (relatively) extensively used to treat diabetic wound patients; however, consistent and rapid healing has yet to be achieved. Some of the latest clinical evidence indicates that there is a wide healing duration range (from 11 days to 6 months) in diabetic wounds after using honey. To reduce this discrepancy, it is important to investigate the best honey composition that is most suitable for treating diabetic wounds even though there are already a few standardized honeys approved for wound care [138]. Therefore, more studies are needed to determine the characteristics present in honey to be termed “standard to treat against diabetic wounds.” The factors that affect honey standardization include the honey source (monofloral and multifloral honey), honey type (processed or raw honey), honey origin (natural honey, commercial or those sold in the supermarkets), and the type and size of the diabetic wounds under treatment.

## 15. Conclusions

Honey is an alternative medicine that is considered to be a suitable therapy with improved outcomes. It is a cost-effective and safe natural agent with rapid diabetic wound healing capacity. However, additional successful clinical evidence is required with validated laboratory findings to establish honey as one of the most effective alternative topical medicines for treating diabetic wounds.

## Figures and Tables

**Table 1:** Successful clinical evidence of using honey on diabetic wounds (2005–2014).

Number	Age of diabetic patient (years)	Sex	Clinical complications	Type of honey	Course of treatment	Resolution time	Reference (country, year)
1	52	Male	Postoperative (amputation) right foot ulcer	Natural honey	Once daily	4 weeks	[139] (Qatar, 2014)

2	Male: 54.6 ± 12.7 (Mean ± SD)	Male	Wagner grades II, III, and IV diabetic foot ulcers Diabetic foot ulcers	Manuka honey	Once daily	11 days	[130] (Saudi Arabia, 2013)
	Female: 58.7 ± 13.4 (Mean ± SD)	Female					

3	55	Male	Diabetic foot ulcer	Natural honey	Once daily	4 weeks	[140] (Qatar, 2012)

4	48	Male	Postoperative (amputation) right foot wound	Honey-based gel	Twice weekly	2 months	[141] (Portugal, 2011)

5	87	Female	Postoperative (amputation) left foot wound	Honey-based gel	Once daily	4 weeks	http://www.l-mesitran.com/sites/l-mesitran.com/files/c120.pdf (2011)

6	52.3 (Mean) (*n* = 30)	56.7% Male	Diabetic wound	Pure raw untreated commercial honey	Once daily	2.3 weeks (mean)	[142] (Egypt, 2010)

7	46 (Mean) (*n* = 12)	8 Male	Wagner types I, II, III, and IV	Natural honey	Initially once a day	Fast excellent recovery	[143] (Pakistan, 2009)

8	52.1 (Mean) (*n* = 30)	15 Male	Wagner grade II diabetic foot ulcers	Commercial honey	Once daily	14.4 days	[126] (Malaysia, 2008)
		15 Female					

9	65	Male	Diabetic foot wound	A paste made of honey, propolis, and myrrh	Once daily	4 weeks	[131] (Saudi Arabia, 2006)

10	79	Male	Diabetic foot ulcer	Supermarket honey	Once daily	6 months	[144] (USA, 2005)

11	62	Male	Diabetic foot ulcer	Manuka honey	Twice weekly	54% reduced by 4 weeks	[121] (Ireland, 2005)

**Table 2:** The effectiveness of honey against microorganisms that are usually found in diabetic wounds.

Microorganisms found in diabetic wounds and references	Microorganisms found to have sensitivity to honey	Honey origin and type with reference
**Gram-positive aerobes**		
*Staphylococcus* species [14, 145–153], including methicillin-resistant *Staphylococcus aureus* (MRSA), coagulase-negative *Staphylococcus epidermidis *	*Staphylococcus aureus *	Nigeria, unprocessed raw honey [154]
		New Zealand, Pasture and Manuka honey [60]
		United Arab Emirates, Manuka honey [155]
		Saudi Arabia, commercial honey [156]
		USA, commercial honey [157]
	MRSA ATCC 43300, MRSA 00791 and 28965	Rio San Pedro Ltd. Chile, Ulmo 90 honey [158]
	*Staphylococcus aureus* ATCC 25923	Australia, Medihoney [159]
*β*-Hemolytic streptococci [145, 146, 148, 151, 152], including *Streptococcus pyogenes, Streptococcus pneumoniae *	*Streptococcus pyogenes *	Nigeria, unprocessed raw honey [154]
		United Arab Emirates, Manuka honey [155]
*Bacillus* species [145]	*Bacillus cereus*, *Bacillus subtilis *	India, raw local honey [160]
*Corynebacterium *sp. [14]	*Corynebacterium pseudotuberculosis *	Egypt, various monofloral honeys [161]

**Gram-negative aerobes**		
*Escherichia coli* [146–150]	*Escherichia coli *	Nigeria, unprocessed raw honey [154]
		United Arab Emirates, Manuka honey [155]
		India, raw and processed local honey [160]
		Rio San Pedro Ltd. Chile, Ulmo 90 honey [158]
		Australia, Medihoney [159]
		Saudi Arabia, commercial honey [156]
*Proteus* species [14, 146, 147, 149, 150, 153, 162]	*Proteus mirabilis* (indole positive)	Nigeria, unprocessed raw honey [154]
*Klebsiella* species [146, 162]	*Klebsiella pneumoniae *	Nigeria, unprocessed raw honey [154]
		Australia, Medihoney [159]
		Turkey; Anzer, Bayburt and Chest nut honey [163]
*Pseudomonas aeruginosa* [14, 148–150, 153, 162]	*Pseudomonas aeruginosa *	Ethiopia, Raw honey [164]
		India, raw and processed local honey [160]
		Rio San Pedro Ltd. Chile, Ulmo 90 honey [158]
		India, local marketed honey [165]
		Australia, Medihoney [159]
		Saudi Arabia, commercial honey [156]

**Anaerobes**		
*Bacteroides* species [14, 146–148, 153]	*Bacteroides fragilis *	Nigeria, unprocessed raw honey [154]
*Clostridium* species [146, 147]	*Clostridium welchii, Clostridium tetani *	Nigeria, unprocessed raw honey [154]
*Peptostreptococcus* species [146, 148]	*Peptostreptococcus *	Thailand, commercial honey [108]
*Enterococci* [14, 147, 148]	*Enterococcus faecalis *	Nigeria, unprocessed raw honey [154]
		Australia, Medihoney [159]
*Prevotella* species [148]	*Prevotella intermedia, Prevotella nigrescens *	Brazil, propolis [166]
*Porphyromonas* species [148]	*Porphyromonas gingivalis *	New Zealand, Manuka honey [167]

**Fungus**		
*Candida tropicalis* [149, 150]	*Candida tropicalis *	Iran, local honey [168]
*Candida albicans* [150]	*Candida albicans *	Nigeria, unprocessed raw honey [154]
		United Arab Emirates, Manuka honey [155]
		Iran, local honey [168]

**Table 3:** Limitations of common topical agents used in wound healing.

Name	Side effects and limitation	References
Silver nitrate	(1) Skin discoloration and irritation	[169]
	(2) Toxicity to epithelium	[170]
	(3) The bacterial reduction of nitrate to nitrite may lead to methemoglobinemia with the use of this topical agent	[171]

Silver	Absorption, systemic distribution, and excretion in urine	[169]

Polyhexamethylene biguanide	Ineffective when there is a measurable degree of wound fluid suppuration because of its short residence times on the wound site	[172]

Proflavine	Induces mutations in bacterial and cell cultures	[173]

Povidone iodine	(1) Short residence times on the wound site	[172]
	(2) Can cause severe metabolic acidosis	[174]
	(3) Cytotoxicity against leukocytes, fibroblasts, and keratinocytes	[175]
	(4) Polymorphonuclear leukocytes are inhibited by this topical agent	[176]
	(5) Povidone iodine has also reportedly been inactivated by wound exudates	[177]
	(6) May “harden” wound eschar rather than softening it, thus increasing the difficulty and discomfort of wound debridement	[178]
	(7) Should not be used during pregnancy, on a newborn, on small children, or on patients with suspected or known thyroid disease	[179]

Hydrogen peroxide	(1) Formation of air emboli in wounds	[180]
	(2) The mechanical cleansing effect of hydrogen peroxide, often attributed to its “fizzing” (which is caused by its decomposition into oxygen and water when it comes in contact with blood and tissue fluids), is questionable	[181]
	(3) Is toxic to fibroblasts	[182]
	(4) Impairs the microcirculation of wounds	[183]
	(5) Limited bactericidal effectiveness	[182]

Diluted iodine solutions (iodine solution USP [United States Pharmacopeia] [2% iodine, 2.5% sodium iodide] and Iodine tincture USP [2% iodine, 2.5% sodium iodide, 50% alcohol])	May irritate tissue, stain the skin, and cause sensitization	[184]

Povidone iodine	Contact dermatitis has been reported with prolonged uninjured skin exposure to ointment	[185]

Chlorhexidine	(1) Associated with few adverse effects on healing	[175]
	(2) MRSA resistance has been found	[186]

Chlorhexidine gluconate solution	Prolonged, repeated use may lead to contact dermatitis	[187]

Acetic acid 0.5%.	(1) Acetic acid has demonstrated toxicity to fibroblasts in culture	[188]
	(2) Reduced epithelial cell proliferation in culture	[189]
	(3) Delayed healing of cultured epithelial autografts has been reported at 0.25% strength	[190]
	(4) Acetic acid has been shown to reduce PMN function	[176]

Sodium hypochlorite (Dakin's solution)	(1) Has caused toxicity to fibroblasts in culture	[170]
	(2) Toxicity to keratinocytes in culture	[170]
	(3) Polymorphonuclear leukocyte viability is inhibited	[176]
	(4) Acidosis may result from continuous use over large-area wounds. This solution may also cause pain	[178]

**Topical pharmaceutical semisolid formulations (ointments and creams)**		
Silver sulfadiazine cream	(1) Not effective for highly exuding wounds; rapidly absorbs fluid, loses its rheological characteristics, and becomes mobile as it remains on wounds for longer periods of time	[172]
	(2) 3–5% incidence of reversible leucopenia	[191]
	(3) There is evidence that silver sulfadiazine is toxic to human keratinocytes and fibroblasts in vitro	[170]
	(4) Should be avoided during pregnancy, on premature infants or on infants younger than 2 months of age	[192]

Silver nitrate ointment	Same problems as silver sulfadiazine cream	[172]

Neosporin	Hypersensitivity is more common because of the presence of neomycin in the ointment	[178]

Nitrofurazone 0.2% compound	(1) Bacteria may develop mild resistance with prolonged use	[178]
	(2) Detrimental effects on the growth and migration of keratinocytes in culture	[170]
	(3) The development of the usual symptoms of contact dermatitis (rash, local edema, and pruritus)	[178]

Gentamicin 0.1% cream	(1) Can inhibit PMN activity	[176]
	(2) Skin hypersensitivity has been reported	[178]
	(3) Ototoxicity and nephrotoxicity can occur, particularly when used in large volumes or for an extended period of time	[193]

Mafenide acetate 0.5% cream (Sulfamylon)	(1) Inhibits human keratinocytes and fibroblasts in vitro	[170]
	(2) Mafenide suppresses PMN and lymphocyte activity	[31, 51]
	(3) The chance of a sulfa allergy is higher with mafenide acetate, and rashes may be seen in approximately *50%* of patients receiving mafenide treatment	[177]
	(4) Toxicity may increase in correlation with the duration of treatment and size of the treated area	[194]
	(5) Painful upon application	[195]

Neomycin ointment	(1) Hypersensitivity reactions, particularly skin rashes, also occur more frequently with neomycin (occurring in 5%–8% of patients)	[196]
	(2) Ototoxicity and nephrotoxicity have been reported for large wounds	[193]
